# Correlation Between Biomarkers and Age-Adjusted Charlson Comorbidity Index in Patients With COVID-19: A Cross-Sectional Study in a Tertiary Care Center in South India

**DOI:** 10.7759/cureus.36000

**Published:** 2023-03-10

**Authors:** Surupa S Kurien, Regi David, Ravi P Varma, Anaga S Dev, Ajitha Chellappan, IP Yadev

**Affiliations:** 1 Pathology and Laboratory Medicine, Government Medical College & Hospital, Thiruvananthapuram, Thiruvananthapuram, IND; 2 Internal Medicine, Government Medical College & Hospital, Thiruvananthapuram, Thiruvananthapuram, IND; 3 Achutha Menon Centre for Health Science Studies, Sree Chitra Tirunal Institute for Medical Sciences and Technology, Thiruvananthapuram, IND; 4 Internal Medicine, Government Medical College & Hospital, Konni, Pathanamthitta, IND; 5 General Surgery, Government Medical College & Hospital, Kollam, Kollam, IND

**Keywords:** meta-inflammation, covid-19, india, biomarkers, comorbidity, age-adjusted charlson comorbidity index

## Abstract

Background

Coronaviruses, generally known to cause a mild degree of respiratory illness have in the recent past caused three serious disease outbreaks. The world is yet to be released from the grip of the most recent coronavirus disease 2019 (COVID-19) pandemic due to emerging mutant strains. Age, presence of comorbidities, clinical severity, and laboratory markers such as C-reactive protein and D-dimer are some of the factors being employed to prioritize patients for hospital care. It is known that comorbidities themselves are an outcome of inflammation and can induce a pro-inflammatory state. Our study aims to elucidate the influence of age and comorbidities on laboratory markers in patients with COVID-19.

Methodology

This is a single-center retrospective study of patients with a laboratory diagnosis of COVID-19 admitted to our hospital between September 21, 2020, and October 1, 2020. A total of 387 patients above the age of 18 years were included in the analysis and categorized based on the age-adjusted Charlson comorbidity index (ACCI) score into group A (score ≤4) and group B (score >4). Demographic, clinical, and laboratory factors as well as outcomes were compared.

Results

Group B exhibited higher intensive care unit admission and mortality, as well as statistically significant higher mean values of most laboratory markers. A correlation was also observed between the ACCI score and biomarker values. On comparison between the two groups regarding cut-offs predicting mortality for laboratory determinants, no consistent pattern was observed.

Conclusions

A correlation between age, the number of comorbidities, and laboratory markers was observed in our analysis of COVID-19-affected patients. Aging and comorbid conditions can produce a state of meta-inflammation and can thereby contribute to hyperinflammation in COVID-19. This can be an explanation for the higher risk of COVID-19-related mortality in older individuals and those with underlying comorbidities.

## Introduction

Coronaviruses are a group of viruses that generally cause a mild degree of respiratory disease [1]. However, the last two decades saw three disease outbreaks by coronaviruses causing severe to fatal disease, namely, SARS-CoV causing severe acute respiratory syndrome (SARS), MERS-CoV causing Middle East respiratory syndrome (MERS), and SARS-CoV-2 leading to coronavirus disease 2019 (COVID-19) [2]. The last one has evolved into a pandemic that has gripped the world since the first outbreak in the Hubei province of China in December 2019. The novel coronavirus infection that was declared a pandemic by the World Health Organization on March 11, 2020, has infected both high and low-income countries alike. However, the brunt of it was borne by countries with limited health resources given the explosive nature of the pandemic [3]. The world is still grappling with the pandemic. Mutant strains have prevented the reining in of the outbreak [4].

Much of the research on the previous two coronavirus outbreaks has contributed immensely toward guiding research and management of the novel coronavirus infection [5]. It is imperative that we continue our quest for triaging as well as refining the management of patients with COVID-19, which will surely be an asset for similar challenges in the future.

During the current pandemic, it was evident that regions with limited resources needed to categorize and prioritize patients for optimal use of health resources and better delivery of healthcare. In this endeavor, several risk factors of COVID-19-related mortality were identified. This included demographic and clinical factors as well as laboratory parameters [6-8]. C-reactive protein (CRP) and D-dimer are some of the biomarkers that have been used as predictors of mortality in triaging patients globally [8]. Age and the presence of comorbidities are other known risk factors for COVID-19-related mortality [8]. However, the effect of the presence of comorbidities on these laboratory parameters has not yet been extensively unraveled.

Hyperinflammation is a key event in COVID-19-affected patients with adverse outcomes. However, comorbidities themselves can independently induce a pro-inflammatory state with the derangement of laboratory markers such as acute-phase reactants [9,10]. Additionally, research has shown inflammation to be an underlying risk factor for morbidities such as cardiovascular disease, diabetes, and cancer [10]. Moreover, studies have also demonstrated that increased levels of inflammatory markers can predict the development of these diseases. The National Survey of Mid-life in the United States showed that circulating levels of interleukin 6 (IL-6) and CRP increased with increasing numbers of chronic conditions [11]. Shaheer et al. reported elevated levels of serum high-sensitivity CRP in patients with type 2 diabetes mellitus and diabetic nephropathy in comparison with non-diabetic controls [12]. Similarly, age is also known to influence the levels of many of these laboratory predictors even in the absence of COVID-19. This is attributed to chronic low-grade inflammation associated with aging also known as inflammaging [13]. This calls for the need to take into account age and the presence of comorbidities while analyzing laboratory values in COVID-19 patients.

Some studies have combined factors related to comorbidity and laboratory predictors to derive prognostic scores such as the 4C mortality score and CoLACD (CoVID-19 lymphocyte ratio, age, Charlson comorbidity index (CCI) score, dyspnea) score to predict COVID-19 related mortality [14,15]. Eight important predictors of COVID-19-related mortality were taken up for computing the 4C mortality score. This included age, sex, number of comorbidities defined according to the modified CCI, respiratory rate, peripheral oxygen saturation, level of consciousness, urea level, and CRP. The parameters taken into account in the CoLACD score combine lymphocyte percentage, age, CCI, and the presence of dyspnea. A study conducted in Mysore, India explored the effect of the presence of multiple comorbidities on laboratory cut-offs of biomarkers [16]. For this, the study categorized patients according to CCI and compared both the biomarker values as well as the cut-offs between the different categories.

CCI that combines 19 parameters is a long-used validated score for assessment of the presence of multiple comorbidities in a patient to predict disease outcome [17]. During the current pandemic, it has been shown to be a useful predictor of mortality in COVID-19-affected patients [18]. Age-adjusted Charlson comorbidity index (ACCI) score, a modified score with age as an additional index, has also been shown to be a similar predictor of adverse disease outcomes in patients with COVID-19 [19]. In our current analysis, we attempted to explore the correlation between comorbidities and laboratory parameters in COVID-19 by employing ACCI.

## Materials and methods

This is a single-center, record-based, cross-sectional study conducted in Government Medical College Hospital, Thiruvananthapuram, which is a public sector tertiary care center and teaching hospital. The study was conducted after obtaining ethical clearance from the Institutional Ethics Committee (04/87/2020/MCT). The study population was laboratory-confirmed consecutive cases of COVID-19 admitted to our hospital between September 21, 2020, and October 1, 2020. We had determined the laboratory predictors of COVID-19-related mortality in our earlier analysis, the detailed methodology and findings of which have been published [20]. For the current analysis, we further categorized patients according to the ACCI score, as shown in Table 1.

**Table 1:** Age-adjusted Charlson comorbidity index score.

Score	Comorbidity
1	For each decade over 40 years add 1 point up to a maximum of 4 points for the age group 71 or older
1	Myocardial infarction, congestive heart failure, peripheral vascular disease, cerebral vascular disease, dementia, chronic obstructive pulmonary disease, connective tissue disease, peptic ulcer disease, mild liver disease, diabetes without end-organ damage
2	Hemiplegia, moderate/severe renal disease, diabetes with end-organ damage, any solid tumor without metastasis, leukemia, lymphoma
3	Moderate/severe liver disease
6	Metastatic solid tumor, acquired immunodeficiency syndrome

**Figure 1:**
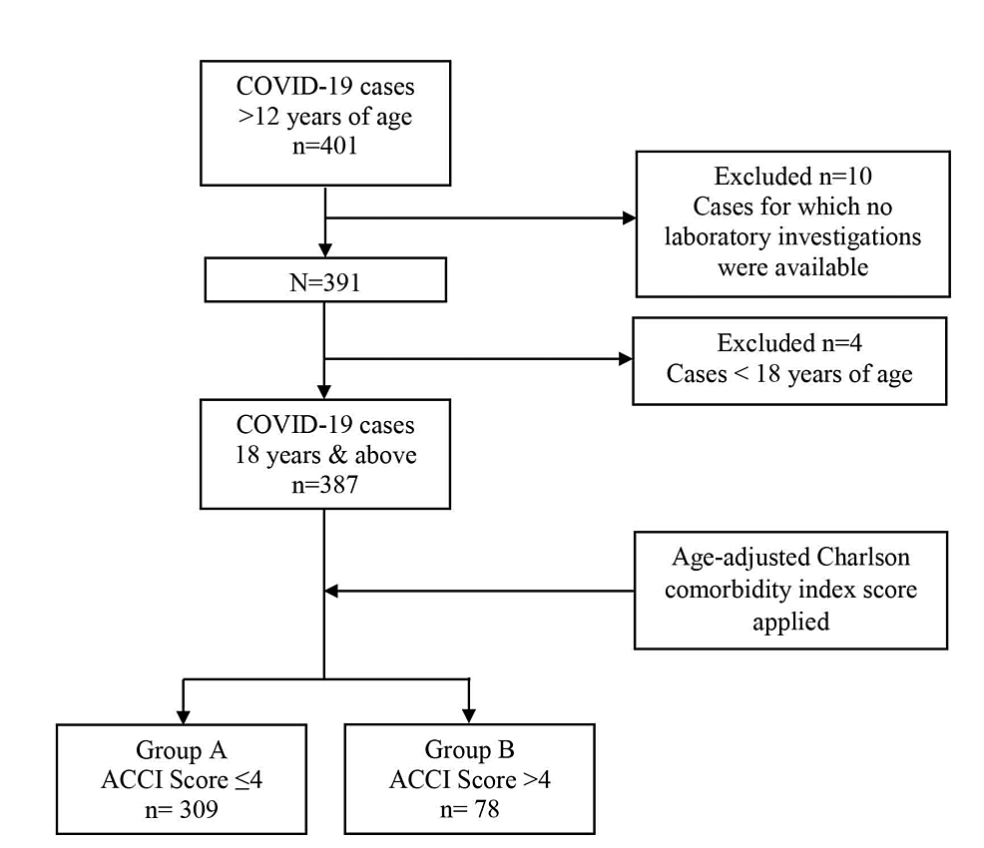
Strobe flowchart. ACCI: age-adjusted Charlson comorbidity index

A total of 387 patients aged 18 years and above were included in the present analysis. The ACCI score was calculated by assigning scores for age and 19 medical comorbidities as per the data obtained from case records. Further, the score obtained was used to classify patients into two groups, namely, group A (score ≤4) and group B (score >4) [19]. See the Strobe flowchart in Figure 1.

Subsequently, we tabulated the demographic features and laboratory findings at the time of admission as well as outcomes in the two groups. For the present analysis, we additionally calculated the derived neutrophil-to-lymphocyte ratio (dNLR), platelet-to-lymphocyte ratio (PLR), and blood urea nitrogen-to-serum albumin ratio (BAR) from the prior laboratory results.

Statistical analysis

Statistical analysis was performed using SPSS for Windows version 25.0 (IBM Corp., Armonk, NY, USA). Continuous variables were expressed as mean and standard deviation and categorical variables as proportions. For univariate analyses, an unpaired t-test was performed for continuous variables and the chi-square test for categorical variables. Spearman’s correlation was performed to explore the relationship between biomarker values and the ACCI score.

Potential cut-off values were calculated in both groups for laboratory parameters that were found to be mortality predictors in our earlier analysis. For each group, we performed receiver operating characteristic analysis (ROC) to explore the ability of each biomarker to classify survivors and non-survivors using a cut-off value. We generated ROC curves, the area under the curve (AUC), p-values, and coordinate points of the curve. Youden’s J was computed for each coordinate point and the point with the highest J value was listed as the optimal cut-off predicting mortality. The sensitivity and specificity of respective cut-off points were also reported.

## Results

There were a total of 309 patients in the lower ACCI group (group A) and 78 in the higher ACCI group (group B).

Table 2 shows the demographic characteristics, severity, and mortality in each of the study groups. Group B had a significantly higher mean age and larger proportion of males when compared to group A. Similarly, higher intensive care unit (ICU) admission and mortality were seen in group B.

**Table 2:** Comparison of demographic features and outcomes between group A and group B. ICU: intensive care unit

		Group A (n = 309 )	Group B (n = 78)	P-value
Age in years	Mean (SD)	49.3 (15.2)	70.4 (10.5)	<0.001
Sex	Male	156 (50.5%)	49 (62.8%)	0.051
	Female	153 (49.5%)	29 (37.2%)	
ICU admission	Yes	47 (15.2%)	30 (38.5%)	<0.001
	No	262 (84.8%)	48 (61.5%)	
Mortality	Died	27 (8.7%)	33 (42.3%)	<0.001
	Survived	282 (91.3%)	45 (57.7%)	

Table 3 presents the distribution (mean and standard deviation) of the biomarkers in group A in comparison to group B. Most biomarkers showed a statistically significant difference in mean between the two groups except for platelet count. Of these biomarkers, mean values of total Leukocyte count (TLC), absolute neutrophil count (ANC), neutrophil-to-lymphocyte ratio (NLR), dNLR, PLR, CRP, ferritin, lactate dehydrogenase (LDH), D-dimer, blood urea nitrogen (BUN), serum creatinine, BAR, and prothrombin international normalized ratio (PT-INR) were higher in group B in comparison to group A while the mean values of absolute lymphocyte count (ALC) and serum albumin were lower in group B.

**Table 3:** Comparison of laboratory parameters between group A and group B. TLC: total leukocyte count; ANC: absolute neutrophil count; ALC: absolute lymphocyte count; NLR: neutrophil-to-lymphocyte ratio; dNLR: derived neutrophil-to-lymphocyte ratio; PLR: platelet-to-lymphocyte ratio; CRP: C-reactive protein; LDH: lactate dehydrogenase; BUN: blood urea nitrogen; BAR: blood urea nitrogen-to-serum albumin ratio; PT-INR: prothrombin time international normalized ratio

	Group A (n = 309)	Group B (n = 78)	P-value
TLC (×10^9^/L)	7.68 (4.55)	10.14 (6.80)	<0.001
ANC (×10^9^/L)	5.54 (4.39)	8.61 (6.81)	<0.001
ALC (×10^9^/L)	1.99 (0.96)	1.46 (0.84)	<0.001
NLR	3.7 (3.8)	8.8 (12.7)	<0.001
dNLR	3.5 (3.7)	8.6 (12.7)	<0.001
Platelet count (×10^9^/L)	220 (90)	210 (100)	0.405
PLR	140.0 (96.5)	209.3 (182.2)	<0.001
CRP (mg/L)	60 (94)	115 (128)	<0.001
Ferritin (ng/mL)	381.3 (433.6)	514.3 (493.3)	0.033
LDH (U/L)	537.2 (427.1)	687.9 (474.3)	0.041
D-dimer (μg/mL)	1.3 (1.9)	3.1 (3.9)	<0.001
PT-INR	1.0 (0.2)	1.2 (0.4)	<0.001
BUN (mg/dL)	32.9 (31.7)	91.6 (83.6)	<0.001
Creatinine (mg/dL)	1.1 (1.8)	3.0 (3.5)	<0.001
Albumin (g/dL)	3.8 (2.1)	3.1 (0.7)	0.010
BAR (mg/g)	9.5 (9.6)	33.2 (34.9)	<0.001

Table 4 shows the correlation of the biomarkers with the ACCI score of the study population. There was a significant correlation between the biomarkers and the ACCI score. All biomarkers except ALC and serum albumin showed a positive correlation with the ACCI score. Both ALC and serum albumin showed a downward trend in values with increasing ACCI scores.

**Table 4:** Correlation between age-adjusted Charlson comorbidity index scores and laboratory parameters. TLC: total leukocyte count; ANC: absolute neutrophil count; ALC: absolute lymphocyte count; NLR: neutrophil-to-lymphocyte ratio; dNLR: derived neutrophil-to-lymphocyte ratio; PLR: platelet-to-lymphocyte ratio; CRP: C-reactive protein; LDH: lactate dehydrogenase; BUN: blood urea nitrogen; BAR: blood urea nitrogen-to-serum albumin ratio; PT-INR:prothrombin time international normalized ratio

	Spearman’s correlation with ACCI score	P-value
TLC	0.207	<0.001
ANC	0.288	<0.001
ALC	-0.274	<0.001
NLR	0.385	<0.001
dNLR	0.384	<0.001
PLR	0.190	<0.001
CRP	0.373	<0.001
Ferritin	0.230	<0.001
LDH	0.232	<0.001
D-dimer	0.408	<0.001
PT-INR	0.160	0.022
BUN	0.523	<0.001
Creatinine	0.342	<0.001
Albumin	-0.468	<0.001
BAR	0.556	<0.001

We classified and compared the optimal biomarker cut-off predicting mortality between the two groups. The cut-off values for each laboratory test for group A and group B are presented in Table 5 together with the AUC, sensitivity, and specificity of each cut-off value.

**Table 5:** Comparison of the optimal biomarker cut-off predicting mortality between group A and group B. ACCI: age-adjusted Charlson comorbidity index; ANC: absolute neutrophil count; CRP: C-reactive protein; BUN: blood urea nitrogen; AUC: area under the curve

	ACCI category	AUC	P-value	Cut-off	Sensitivity	Specificity
ANC	Group A	0.752	<0.001	8.01 (×10^9^/L)	55.6	87.1
	Group B	0.695	0.005	7.39 (×10^9^/L)	63.3	74.4
CRP	Group A	0.840	<0.001	58.6 (mg/L)	82.6	80.0
	Group B	0.796	<0.001	87.5 (mg/L)	68.0	83.3
D-dimer	Group A	0.811	<0.001	1.05 (μg/mL)	82.6	74.4
	Group B	0.786	<0.001	1.01 (μg/mL)	91.3	60.5
BUN	Group A	0.806	<0.001	31.5 (mg/dL)	77.8	74.1
	Group B	0.789	<0.001	52.5 (mg/dL)	82.1	68.2

**Figure 2:**
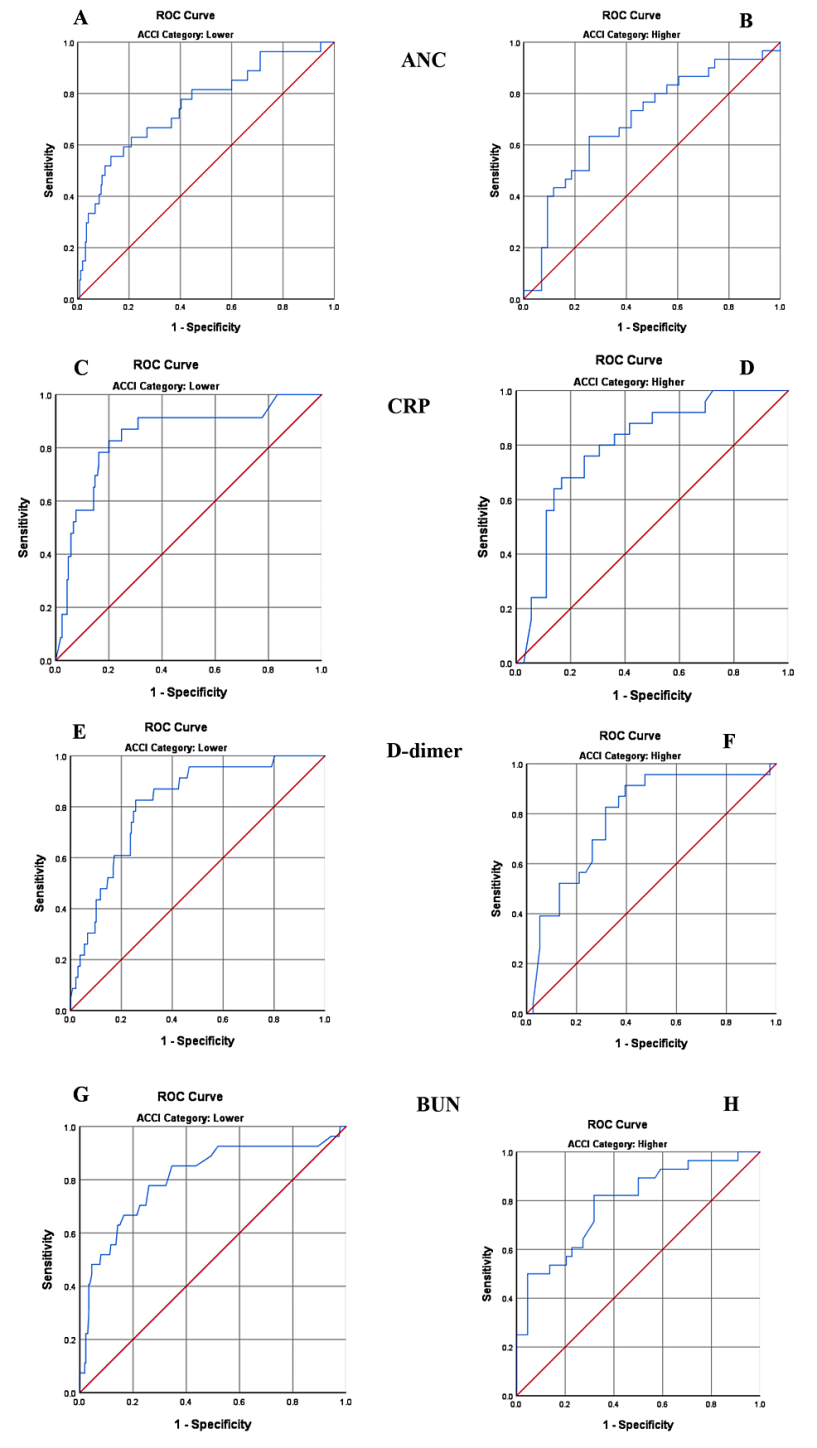
Receiver operating characteristic curve analysis for selected biomarkers. A and B illustrate ANC (group A and group B), C and D illustrate CRP (group A and group B), E and F illustrate D-dimer (group A and group B), and G and H illustrate BUN (group A and group B). ANC: absolute neutrophil count; CRP: C-reactive protein; BUN: blood urea nitrogen; AUC: area under the curve

In our earlier analysis, ANC, CRP, D-dimer, and BUN were shown to be independent predictors of COVID-19-related mortality [20]. Cut-off values predicting mortality were derived for the same biomarkers in the present analysis in both group A and group B. ANC and D dimer showed lower cut-off values in group B when compared to group A while CRP and BUN demonstrated a higher cut-off in group B. Figure 2 illustrates the ROC analysis for selected biomarkers.

## Discussion

Of the total 387 patients included in the analysis, 309 had an ACCI score of ≤4 (group A) and 78 had a score of >4 (group B). Group B showed a higher mean age and proportion of males. This group also exhibited a higher proportion of ICU admission as well as mortality indicating increased disease severity. This demonstrates that the number of comorbidities and age has a significant influence on disease severity and progression. This is in line with earlier studies that have shown both ACCI as well as CCI to be predictors of adverse disease outcomes in COVID-19 patients [18,19].

The mean values of laboratory markers such as TLC, ANC, NLR, dNLR, PLR, CRP, ferritin, LDH, D-dimer, BUN, serum creatinine, BAR, and PT-INR showed a significant difference between the two groups with higher values in group B. Mean values of ALC and albumin were significantly lower in group B. There was also a significant correlation between the laboratory values and the ACCI score. These findings indicate that the number of comorbidities and age significantly influence the serum levels of biomarkers in patients with COVID-19. This is consistent with the findings of Parthasarathy et al. who showed a higher impairment of laboratory determinants in the group with more number of comorbidities [16]. They also reported a similar positive correlation between the laboratory parameters and the CCI score. Elevated neutrophil count, NLR, and dNLR point toward a rapid innate immunity with subsequent hyperactivation of neutrophils and monocytes leading to cytokine storm while lymphopenia indicates a dysregulated T-cell response and suppressed adaptive immunity [21,22]. Similarly, elevated CRP, LDH, and ferritin are all markers of the inflammatory process in COVID-19. It has been shown that multimorbidities themselves are a consequence of inflammation with the elevation of inflammatory markers. The statistically significant increase in serum levels of inflammatory markers in group B supports the hypothesis that pre-existing chronic low-grade inflammation due to comorbid diseases might have a cascading effect in COVID-19 and consequent adverse outcomes.

On exploring the cut-offs between group A and group B for the biomarkers that were shown to predict mortality in the study population, ANC and D-dimer showed a lower cut-off in the group with higher ACCI score (group B) in comparison to the group with lower ACCI score (group A) while CRP and BUN showed a higher cut-off. Parthasarathy et al. in their study had shown that TLC, ANC, NLR, dNLR, and CRP had a consistently lower cut-off in the group with more comorbidities. They employed the CCI score for patient categorization. However, our analysis did not demonstrate such consistent results between groups across all the selected biomarkers. The reasons for this discrepancy are manifold. Because ours is a referral tertiary care center, moderately or severely ill constituted a large proportion of the inpatients, and laboratory derangements were seen in patients belonging to both ACCI categories. Hypertension and obesity, both of which can induce a state of meta-inflammation, are not part of the ACCI score [23,24]. This is one of the limitations of our study. In fact, hypertension was the second highest comorbidity in our study population [20]. Other limitations that could have affected the results are the smaller sample size and the retrospective nature of our study.

## Conclusions

COVID-19, otherwise a mild infection, can be catastrophic in some individuals, with comorbidity and age being significant risk factors. Chronic low-grade inflammation seems to be a contributory factor in this scenario. As the evaluation of laboratory markers holds a key to this, we recommend exploring the cut-off for biomarkers by classifying patients using a validated comorbidity score in a multicentric study with a larger sample size. We believe that hypertension and obesity need to be incorporated in scoring for comorbidity in COVID-19 patients as both can produce a state of meta-inflammation.
